# FSH Regulates YAP-TEAD Transcriptional Activity in Bovine Granulosa Cells to Allow the Future Dominant Follicle to Exert Its Augmented Estrogenic Capacity

**DOI:** 10.3390/ijms232214160

**Published:** 2022-11-16

**Authors:** Leonardo Guedes de Andrade, Valério Marques Portela, Esdras Corrêa Dos Santos, Karine de Vargas Aires, Rogério Ferreira, Daniele Missio, Zigomar da Silva, Júlia Koch, Alfredo Quites Antoniazzi, Paulo Bayard Dias Gonçalves, Gustavo Zamberlam

**Affiliations:** 1 Centre de Recherche en Reproduction et Fertilité, Faculté de Médecine Vétérinaire, Université de Montréal, Saint-Hyacinthe, QC J2S 7C6, Canada; 2 Laboratory of Biotechnology and Animal Reproduction (BioRep), Veterinary Hospital, Federal University of Santa Maria (UFSM), Santa Maria 97105-900, Brazil; 3 Department of Animal Science, Santa Catarina State University (UDESC), Chapecó 88035-901, Brazil; 4 Molecular and Integrative Physiology of Reproduction Laboratory (MINT), Federal University of Pampa (Unipampa), Uruguaiana 97501-970, Brazil

**Keywords:** ovary, cow, Hippo, steroidogenesis, follicle deviation or divergence, yes-associated protein 1, CTGF, CYR61, ANKRD1

## Abstract

The molecular mechanisms that drive the granulosa cells’ (GC) differentiation into a more estrogenic phenotype during follicular divergence and establishment of follicle dominance have not been completely elucidated. The main Hippo signaling effector, YAP, has, however, emerged as a potential key player to explain such complex processes. Studies using rat and bovine GC demonstrate that, in conditions where the expression of the classic YAP-TEAD target gene tissue growth factor (*CTGF*) is augmented, *CYP19A1* expression and activity and, consequently, estradiol (E2) secretion are reduced. These findings led us to hypothesize that, during ovarian follicular divergence in cattle, FSH downregulates YAP-TEAD-dependent transcriptional activity in GC to allow the future dominant follicle to exert its augmented estrogenic capacity. To address this, we performed a series of experiments employing distinct bovine models. Our in vitro and ex vivo experiments indicated that indeed FSH downregulates, in a concentration-dependent manner, mRNA levels not only for *CTGF* but also for the other classic YAP-TEAD transcriptional target genes *ANKRD1* and *CYR61* by a mechanism that involves increased YAP phosphorylation. To better elucidate the functional importance of such FSH-induced YAP activity regulation, we then cultured GC in the presence of verteporfin (VP) or peptide 17 (P17), two pharmacological inhibitors known to interfere with YAP binding to TEADs. The results showed that both VP and P17 increased *CYP19A1* basal mRNA levels in a concentration-dependent manner. Most interestingly, by using GC samples obtained in vivo from dominant vs. subordinate follicles, we found that mRNA levels for *CTGF*, *CYR61*, and *ANKRD1* are higher in subordinate follicles following the follicular divergence. Taken together, our novel results demonstrate that YAP transcriptional activity is regulated in bovine granulosa cells to allow the increased estrogenic capacity of the selected dominant follicle.

## 1. Introduction

Ovarian follicular development and growth in mammals is a complex and dynamic physiological process that requires the interaction of different hormones and cell signaling pathways [1]. In ruminants, as follicle development progresses, follicles gradually become more and more reliant on gonadotropins, first as gonadotropin-responsive follicles and then as gonadotropin-dependent follicles [2]. During the antral growth stage, the most advanced follicles in the pool emerge concomitantly with the increase in circulating follicle-stimulating hormone (FSH) levels to form what is commonly referred to as the cohort of gonadotropin-dependent follicles. During a certain period of this phase, granulosa cells (GC) present similar elevated proliferative capacity, which allows these follicles to grow at an approximately similar rate, until one follicle is selected for further growth [3,4]. In cattle, as in other monovulatory species such as human and equine, this process is known as follicle selection [5,6]. The moment when the selected follicle continues its growth, while the remaining follicles cease growing, is known as follicle deviation or follicle divergence [7].

Probably the most important characteristic of the dominant follicle is its greater capacity for estradiol (E2) production by its GC. After the wave emergence, E2 content in the follicular fluid of the growing dominant follicle increases at least 20-fold by the day of selection [4,5,6,7,8]. Such augmented steroidogenic capacity is mainly due to the fact that GC differentiate to produce more E2. A key steroidogenic enzyme to this process is cytochrome P450 aromatase family 19 subfamily A member 1 (CYP19A1). At the ruminant GC level, this enzyme can metabolize the theca-derived androgen testosterone into E2 and/or the theca-derived androstenedione into estrone (E1), which will then be metabolized into E2 by another steroidogenic enzyme known as 17β-hydroxysteroid dehydrogenase (HSD17B1) [9,10]. In cattle, as well as in other mammalian species, FSH can be considered one of the primary stimulators of GC CYP19A1 expression [11,12]. Despite years of research, the molecular mechanisms that drive the GC differentiation into a more estrogenic profile during follicular divergence have not been completely elucidated. The Hippo signaling has, however, emerged as a potential key player to explain such complex processes.

The core Hippo pathway consists of a kinase cascade that ultimately regulates the activity of the transcriptional activators yes-associated protein 1 (YAP) and transcriptional co-activator with PDZ-binding motif (TAZ). In a conserved manner, it is known that when Hippo signaling is inactive, YAP/TAZ accumulate in the nucleus and form complexes with numerous transcription factors, notably those of the TEA domain transcription factor (TEAD) family of transcription factors, resulting in the modulation of the transcriptional activity of several target genes, such as the classic tissue growth factor (*CTGF*, also known as *CCN2*), ankyrin repeat domain 1 (*ANKRD1*), and cysteine-rich protein 61 (*CYR61*, also known as *CCN1*). Conversely, when Hippo signaling is activated, YAP/TAZ are phosphorylated (at serine residues S127 and S397 for YAP, and at S89 for TAZ), resulting in their nuclear export to the cytoplasm where they will be retained and/or degraded, therefore compromising YAP/TAZ-dependent transcriptional activity [13,14]. A study employing mouse in vivo and in vitro models reported that timely expression and activation of the Hippo effector YAP in GC is critical for ovarian follicle development [15]. Briefly, it was demonstrated that while an increase of YAP-dependent transcriptional activity promotes mouse GC proliferation, it suppresses GC differentiation and steroidogenesis. Although a physiological correlation between FSH and ANKRD1 or CYR61 has not been reported, a study employing rat GC cultures demonstrated that *Ctgf* mRNA downregulation coincides with FSH-induced GC differentiation [16]. Interestingly, a recent study employing a well-defined bovine in vitro GC model clearly showed that when *CTGF* mRNA levels are increased in this cell type, there is a decrease in *CYP19A1* expression and, consequently, a significant reduction in E2 secretion levels [17]. Taken together, these findings led us to hypothesize that, during ovarian follicle divergence in cattle, FSH downregulates YAP-TEAD-dependent transcriptional activity in granulosa cells to allow the future dominant follicle to launch its augmented estrogenic capacity. To address this, we performed a series of experiments employing bovine in vitro, ex vivo, and in vivo models.

## 2. Results

### 2.1. FSH Downregulates, in a Concentration-Dependent Manner, mRNA Levels for CTGF and Other Classic YAP-TEAD Transcriptional Target Genes

**Figure 1:**
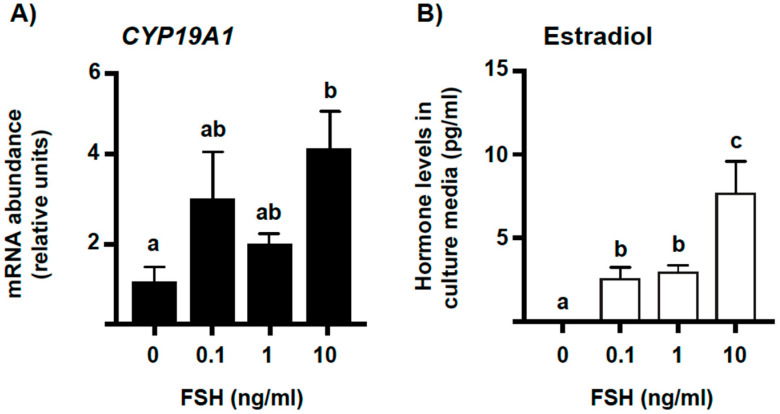
Effect of FSH on *CYP19A1* mRNA abundance and estradiol secretion in vitro. Granulosa cells (GC) were cultured for 6 days under non-luteinizing conditions and treated with graded doses of FSH for the last 4 days of culture (see Section 4 for details). (**A**) Messenger RNA (mRNA) abundance for *CYP19A1* was measured by real-time PCR and normalized to the housekeeping gene *H2AFZ*. (**B**) Estradiol (E2) secretion in culture media was measured by chemiluminescence. Data represent the mean ± SEM for three independent replicate cultures. Bars with different letters are significantly different (*p* < 0.05).

To determine whether FSH regulates the expression of CTGF in bovine granulosa cells, we employed a non-luteinizing GC culture model in which cells were cultured in the presence of graded doses of FSH for the last four days of culture. This is a completely serum-free, long-term GC culture system that allows the induction/maintenance of CYP19A1 expression and E2 secretion in response to physiological doses of FSH [18,19]. As expected, FSH upregulated *CYP19A1* mRNA levels in a concentration-dependent manner (*p* < 0.05, Figure 1A) and, consequently, stimulated E2 secretion in a concentration-dependent manner, too (*p* < 0.05, Figure 1B).

**Figure 2:**
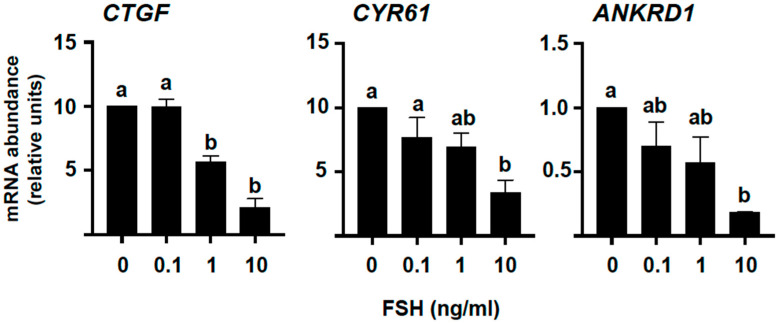
Effect of FSH on mRNA levels for *CTGF* and other classic YAP-TEAD transcriptional target genes in vitro. Granulosa cells (GC) were cultured for 6 days under non-luteinizing conditions and treated with graded doses of FSH for the last 4 days of culture (see Section 4 for details). Messenger RNA (mRNA) abundance for connective tissue growth factor (*CTGF*), ankyrin repeat domain 1 (*ANKRD1*), and cysteine-rich protein 61 (*CYR61*) was measured by real-time PCR and normalized to the housekeeping gene *H2AFZ*. Data represent the mean ± SEM for three independent replicate cultures. Bars with different letters are significantly different (*p* < 0.05).

Conversely, FSH downregulated, in a concentration-dependent manner, the mRNA levels not only for *CTGF* (*p* < 0.05, Figure 2A) but also for *ANKRD1* (*p* < 0.05, Figure 2B) and *CYR61* (also known as *CCN1*; *p* < 0.05, Figure 2B), both considered classic YAP-TEAD target genes along with *CTGF*.

### 2.2. YAP Phosphorylation in Granulosa Cells Increases following FSH Challenge and along the Bovine Follicle Growth

**Figure 3:**
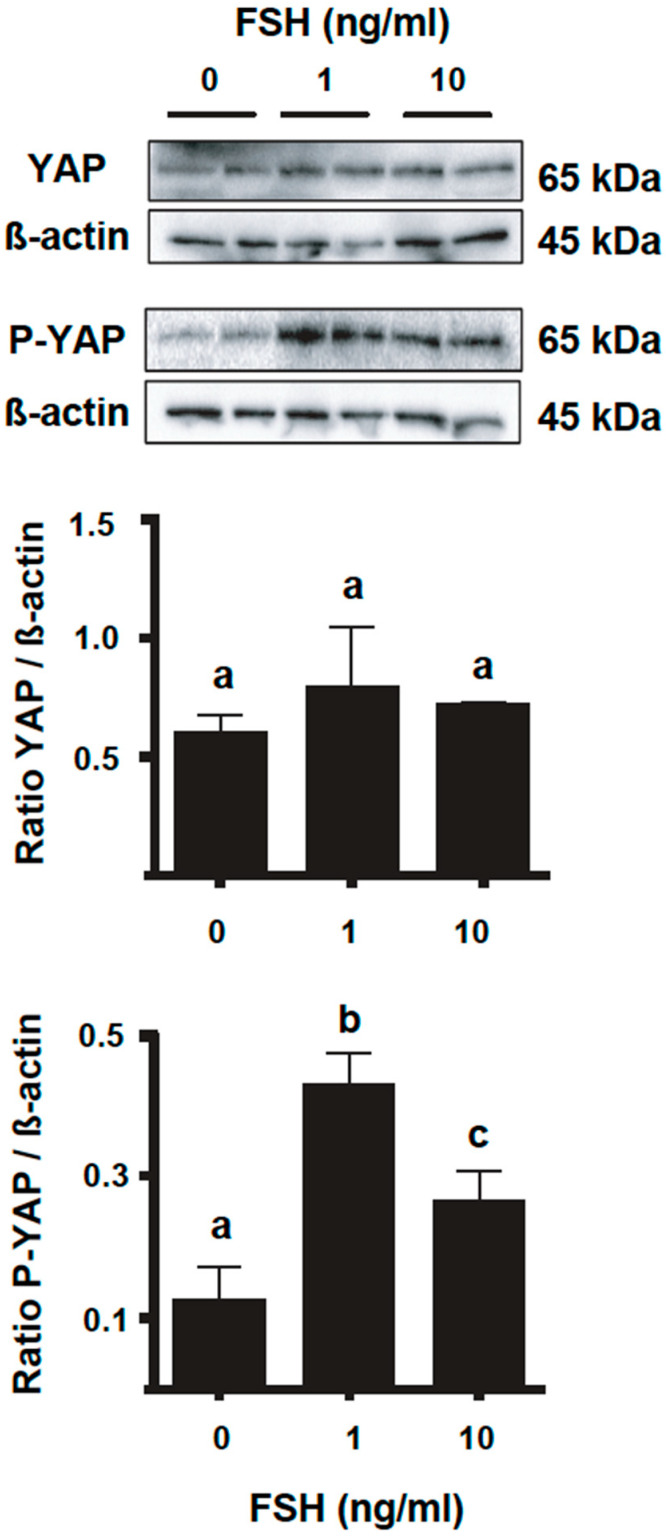
YAP phosphorylation is regulated by FSH in vitro. Granulosa cells (GC) were cultured for 6 days under non-luteinizing conditions and treated with 2 distinct doses of FSH for the last 4 days of culture (see Section 4 for details). Total (YAP) and phosphorylated YAP on serine 127 (P-YAP) protein levels were measured by Western blot and normalized to β-actin, as shown in representative blots (n = 2 replicates). Data are means ± SEM of four independent cultures. Bars with different letters are significantly different (*p* < 0.05).

To determine if the mechanism by which FSH downregulates *CTGF* and the other YAP-TEAD target genes in bovine GC involves regulation of the YAP phosphorylation status, we then cultured cells in the presence of two doses of FSH (1 and 10 ng/mL) for 4 days, and samples were then collected at the end of the culture for Western blot (WB) analyses. The results indicated that FSH treatments did not alter total YAP protein levels (*p* > 0.05, Figure 3) but significantly promoted YAP phosphorylation on serine 127 (Ser127) in a concentration-dependent manner (*p* < 0.05, Figure 3). The phosphorylation of this serine is known to promote a binding site for 14-3-3 protein, leading to YAP-14-3-3 complex formation in the cytoplasm, and therefore affects the binding of this Hippo effector to its target transcription factors at the nucleus [20].

**Figure 4:**
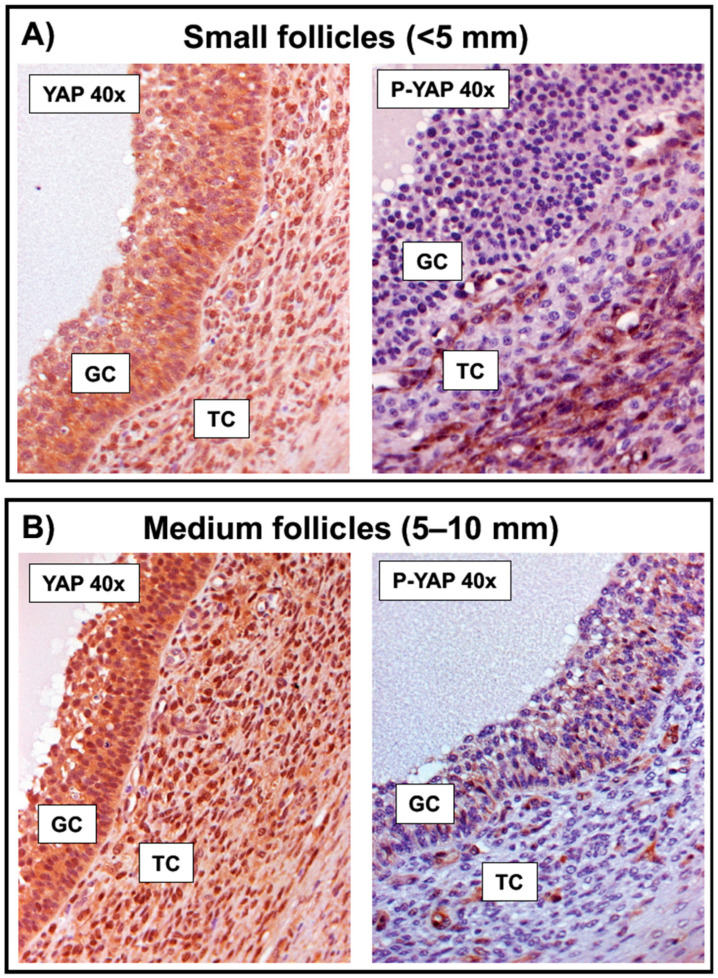
Localization and expression pattern of total (YAP) and phospho-YAP (Ser127) in granulosa cells from follicles of increasing size. Representative immunohistochemistry (IHC) micrographs (objective 40x) show the expression pattern of total (YAP) and phosphorylated YAP on serine 127 (P-YAP) in granulosa (GC) and theca cells (TC) from follicles of (**A**) small (<5 mm) and (**B**) medium (5–10 mm) sizes. While brown color represents a positive immunostaining signal for total and/or P-YAP (detected in nucleus and/or cytoplasm of GC), the counterstain is hematoxylin, which stains the cell nuclei in blue, contrasting with the brown.

To determine if the above-observed in vitro FSH-induced YAP phosphorylation pattern could be somehow observed in GC along the follicle growth, we then collected bovine ovarian follicles of different sizes (compatible with those found in emergence to dominance) for evaluation. The intensity of staining observed suggested that the total YAP expression pattern is similar in GC collected from small (<5 mm) and medium (5–10 mm) follicles (Figure 4A,B). However, while the positive signal for phospho-YAP (Ser127) was absent or barely detected in GC from small follicles (Figure 4A), the staining was easily observed in the cytoplasm of medium follicles (Figure 4B).

### 2.3. The mRNA Abundance of CTGF, ANKRD1, and CYR61 Is Higher in Subordinate Follicles following the Follicular Divergence

**Figure 5:**
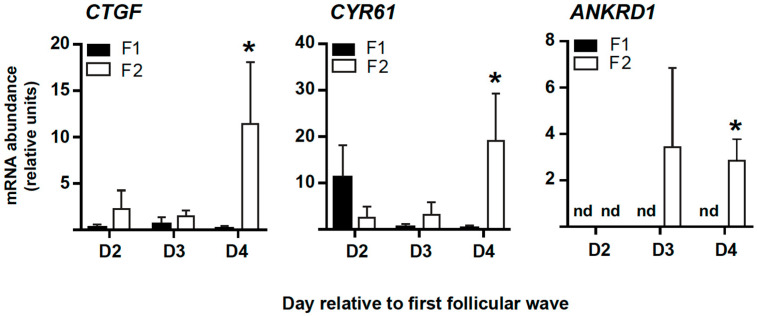
Expression of classic YAP-TEAD transcriptional target genes in granulosa cells during establishment of the dominant follicle in vivo. Granulosa cells (GC) were recovered from the largest (F1—dominant follicles, black bar) and the second largest (F2—subordinate follicles, white bar) follicles collected at days 2 (D2), 3 (D3), and 4 (D4) of the first follicular wave of the synchronized estrous cycle. D2 corresponds to the day before the divergence, D3 corresponds to the day of follicular divergence, and D4 corresponds to the first day after divergence. Messenger RNA abundance for connective tissue growth factor (*CTGF*), ankyrin repeat domain 1 (*ANKRD1*), and cysteine-rich protein 61 (*CYR61*) was measured by real-time PCR and normalized to the housekeeping gene *GAPDH*. Data represent the mean ± SEM of independent follicle samples (n = 4) per group in each time point. An asterisk (*) indicates significant difference between F2 (subordinate) and F1 (dominant) follicle groups over day-matched comparison (*p* < 0.05) and “nd” denotes non-detectable amplification in the real-time PCR analysis.

The previously described in vitro and ex vivo findings suggested that FSH may promote YAP phosphorylation to decrease the expression of CTGF and other classic YAP-TEAD target genes to allow GC from growing follicles to better express their estrogenic capacity and, consequently, establish their dominance over subordinate follicles in the same cohort. To confirm that, an in vivo experiment was then performed to obtain the largest (F1: herein also referred to as dominant follicle) vs. the second largest (F2: herein also referred to as subordinate follicle) GC samples from ovaries collected at days 2 (D2), 3 (D3), and 4 (D4) of the first follicular wave (collection time points correspond to the days before, during, and after ovarian follicular follicle deviation in bovine, respectively). Although mRNA levels for *CTGF*, *CYR61*, and *ANKRD1* were not significantly higher in GC of subordinate follicles collected at D2 and D3 (*p* > 0.05, Figure 5), mRNA levels for those 3 genes were significantly higher in GC from subordinate follicles collected at D4 (*p* < 0.05, Figure 5). The latter day corresponds to the first day after divergence when the dominant follicle has established its dominance and presents much higher mRNA levels for *CYP19A1* in GC, and consequently, higher intra-follicular E2 levels, as it can be confirmed in a previous publication [8].

### 2.4. Pharmacological Inhibition of YAP-TEAD Interaction In Vitro Increases Basal Levels for mRNA Encoding CYP19A1

**Figure 6:**
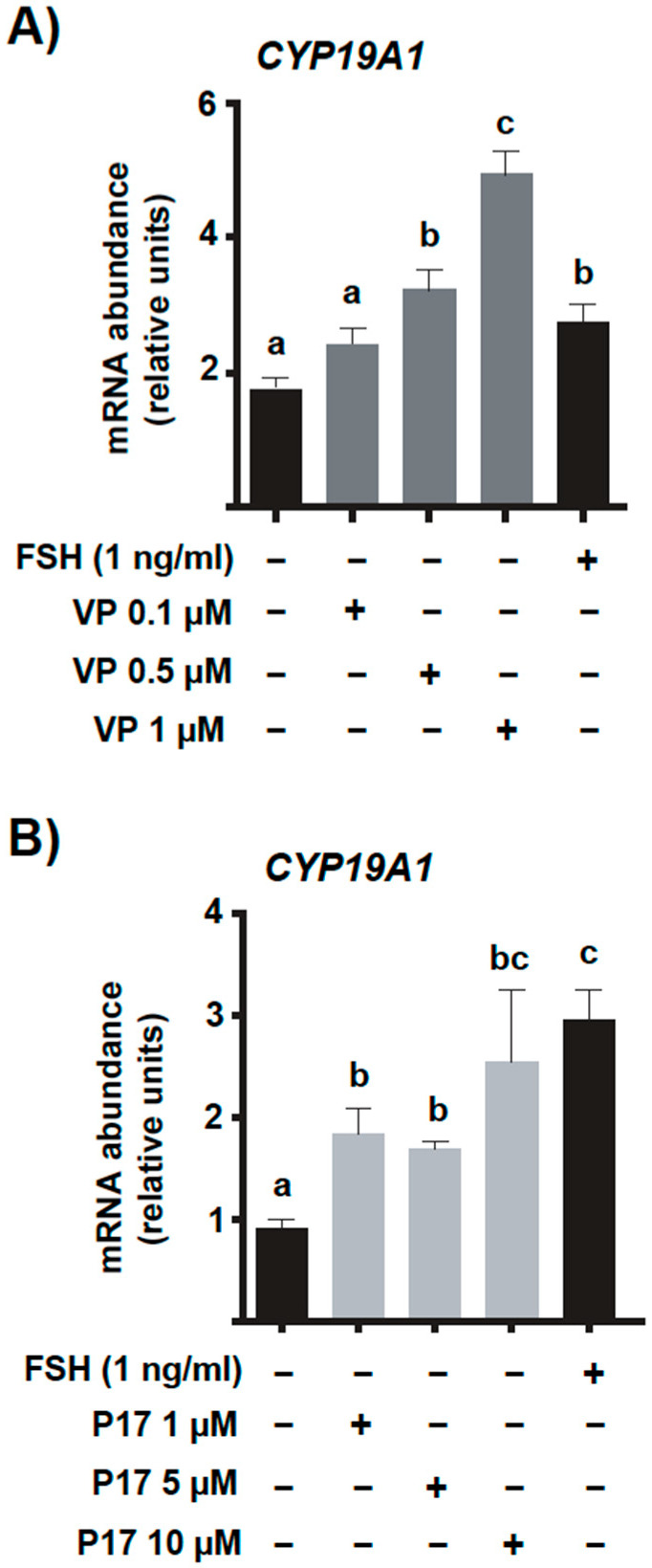
Pharmacological inhibition of YAP-TEAD interaction increases basal *CYP19A1* mRNA abundance in vitro. Granulosa cells (GC) were cultured for 6 days under non-luteinizing conditions (see Section 4 for details) and treated (for the last 4 days of culture) with different concentrations of (**A**) Verteporfin (VP: 0, 0.1, 0.5, and 1 μM) or (**B**) Peptide 17 (P17: 0, 1, 5, and 10 μM), molecules known to interfere with YAP binding to the TEAD family of transcription factors. A FSH treatment group (1 ng/mL) was included from day 2 to 6 in both experiments as a positive control for *CYP19A1* stimulation. Messenger RNA abundance for *CYP19A1* was measured by real-time PCR and normalized to the housekeeping gene *H2AFZ*. Data are means ± SEM of three independent cultures. Bars with different letters are significantly different (*p* < 0.05).

Some of the experiments previously performed in the present study clearly confirmed that, in bovine GC, there is a clear inverse relationship not only between the expression levels of *CYP19A1* and *CTGF*, but also an inverse relationship between *CYP19A1* and other YAP-TEAD classic target genes. To better elucidate the nature of such relationship (cause vs. consequence), we decided to perform a series of in vitro experiments using pharmacological inhibitors known to interfere with YAP binding to TEAD family transcription factors. In the first series of cultures, GC were cultured without or in the presence of different concentrations of verteporfin (VP), a well-known and commonly used YAP-TEAD inhibitor molecule that decreases basal levels of *CTGF*, *CYR61*, and *ANKRD1* in a concentration-dependent manner [21,22]. The results shown herein indicated that VP increased *CYP19A1* basal mRNA levels in a concentration-dependent manner (*p* < 0.05, Figure 6A).

To further investigate whether VP action was specific or not, we then decided to use an alternative inhibitor, peptide 17 (P17), which is an engineered peptide that also disrupts the YAP-TEAD interaction [23,24]. Similar to what was observed for VP, *CYP19A1* basal mRNA levels were also increased in a concentration-dependent manner following P17 treatment (*p* < 0.05, Figure 6B). Together, these findings clearly indicate that CTGF and/or other YAP-TEAD target genes exert a direct or indirect inhibitory effect of *CYP19A1* transcriptional regulation in bovine GC.

## 3. Discussion

The establishment of ovarian follicle dominance in monovulatory species involves complex and dynamic processes. Despite years of research, some key molecular mechanisms that drive the GC differentiation into a more estrogenic phenotype during follicular divergence remain unclear. In the present study, we used bovine in vitro, ex vivo, and in vivo models to generate novel and exciting data, showing an important role of the Hippo effector YAP during the establishment of follicular dominance. Together, our data indicate that YAP transcriptional activity is downregulated in bovine granulosa cells by FSH to allow or facilitate the increased estrogenic capacity of the selected dominant follicle.

In terms of the expression pattern for the Hippo effector YAP in bovine ovarian follicles, a recent study in this species showed via WB analysis that total YAP expression levels were similar in GC isolated from all stages of follicle development (2–5, 5–10, >10 mm) [25]. These authors, however, did not include phospho-YAP (Ser127) in such WB comparison nor did they evaluate the effect of FSH or any other growth factor on total and phospho-YAP protein levels. Although their WB results for total YAP in GC isolated from different follicle sizes corroborate the total YAP expression stability pattern observed in our IHC analysis and following our FSH treatment in vitro, our study clearly contributes with novel data for this and other monovulatory species, particularly related to FSH-induced YAP phosphorylation aspects and its consequence to YAP-TEAD-related transcriptional activity during the establishment of follicle dominance.

FSH action in GC occurs mainly through the adenylyl cyclase (cAMP) pathway [11,26]. Forskolin is a pharmacological agonist of adenylyl cyclase which is widely used to mimic FSH in activating differentiation signaling in GC [27]. Treatment of mouse GC with forskolin induced phosphorylation of YAP protein at serine 127 faster than forskolin increased *Cyp19a1* mRNA abundance in this cell type [15]. In addition, these same authors employed a human ovarian granulosa cell-like tumor cell line (KGN) to show that constitutively transcriptional active YAP (YAPS127A) significantly suppresses E2 production by these cells. These authors, nevertheless, did not assess (in neither their mouse models nor in their human models) any classic YAP-TEAD target genes, nor did they discuss in the latter experiment whether the expression of CTGF, CYR61, or ANKRD1 could be directly or indirectly related to *CYP19A1* transcriptional regulation in GC.

Although a previous study in rat GC has reported that FSH downregulates *Ctgf* in the same conditions that it stimulates E2 secretion [16], such study only showed a negative correlation and never confirmed if CTGF by itself can indeed alter *Cyp19a1* transcription in this cell type. Similarly, a study in bovine GC [17] used the same cell culture system that we employed in the present study to also observe such inverted correlation between CTGF expression and *CYP19A1*/E2 levels in bovine species. Nevertheless, no functional experiment was performed by these authors. To the best of our knowledge, our study shows the first evidence that disrupting the YAP–TEAD interaction, and consequently affecting CTGF expression, leads to basal *CYP19A1* mRNA levels’ augmentation in GC. Such pharmacological inhibition, however, does not alter only *CTGF*, but also affects the basal levels of the other classic YAP-TEAD target genes *ANKRD1* and *CYR61.* Studies showing the physiological roles exerted by these two proteins in bovine ovary are, nevertheless, scarce. While the expression of *ANKRD1* in bovine GC has been associated with a period of decreasing oocyte competence and ANKRD1 was pointed out as a gene that can lead to apoptosis and atresia [28], CYR61 is known to be expressed in bovine granulosa-derived luteal cells and it has been identified as a potential molecular mediator of angiogenesis in the CL [29]. Interestingly, our in vivo experiment showed that indeed not only *CTGF* but also *CYR61* and *ANKRD1* are significantly higher in the largest subordinate follicles at the day after ovarian follicular deviation in bovine was established. Together, our in vivo findings and our in vitro experiments, using pharmacological YAP-TEAD inhibitors and challenging GC with FSH doses, suggest that not only CTGF but other YAP-TEAD-related genes might be involved not only with the transcriptional machinery responsible for *CYP19A1* regulation in GC, but also with the GC differentiation process required for it. The precise effects and respective mechanisms of action of CTGF, CYR61, and ANKRD1 in bovine GC, nevertheless, remain to be further investigated.

Even though our results employing distinct bovine models complement each other’s findings, a puzzling question also remains to be better addressed: does the expression of these YAP-TEAD target genes decrease in GC from the selected dominant follicle, or is the expression of those genes augmented in GC from subordinate follicles during the divergence process? Based on the expression pattern for these genes in dominant vs. subordinate follicles collected at each of the days tested herein (collection time points correspondent to the days before, during, and after ovarian follicular follicle deviation in bovine, respectively), it is most likely that the mRNA levels for these YAP-TEAD targets started increasing in subordinate follicles during the divergence to then be significantly augmented after ovarian follicular deviation was established. This possibility is supported by the fact that fibroblast growth factor 2 (FGF2), a FGF related to bovine follicle atresia, augments *CTGF* mRNA abundance in a dose- and time-dependent manner in bovine GC cultured in vitro [17]. Curiously, FGF2 is known for inhibiting steroidogenesis in bovine GC by suppressing *CYP19A1* expression [30,31]. On the other hand, taking into consideration the facts that FSH increases YAP phosphorylation in bovine GC (data shown herein) and that the future dominant follicle is known for being more responsive to FSH as its circulating levels increase [32,33], it is plausible to suggest that YAP-dependent transcriptional activity is inhibited or, at least, transiently controlled in the selected dominant follicle until it establishes its estrogenic dominance over other follicles from the same cohort. To better understand such puzzle, nevertheless, it is important to take into consideration which main functions are normally attributed to Hippo effectors along the follicle development dynamics in mammals, particularly related to early stages of follicle development.

In murine models, it was demonstrated that induced ovarian fragmentation promotes follicle growth, which is related to decreased phospho-YAP levels, increased nuclear localization of YAP, and consequently, enhanced expression of CTGF [34]. Briefly, ovaries from juvenile mice (containing secondary and smaller follicles) were cut in 3–4 fragments and then allo-transplanted under kidney capsules of adult hosts. Histological analyses and follicle counting of grafts indicated an augmentation in the percentage of late secondary and antral/preovulatory follicles accompanied by decreases in primordial follicles. In addition, these authors also demonstrated that such fragmentation-induced follicle growth was partially blocked by CTGF antibodies or by verteporfin, and that CTGF and CYR61 recombinant proteins promoted the development of primary follicles to the late secondary/antral stage in ovarian explants. In both circumstances, however, the authors attributed a key role to the fact that CCN growth factors (CTGF and CYR61) can promote GC proliferation. Indeed, another study in mice demonstrated that stimulation of YAP-dependent transcriptional activity promotes mouse GC proliferation, however such induction consequently suppresses GC differentiation and steroidogenesis [15]. Curiously, a recent study in mice showed that YAP-induced transcriptional activity in large antral follicles is essential for LH-induced ovulatory cascade [35]. Taken together, these findings in murine models strongly indicate that the expression and activation of the Hippo effector YAP in murine GC may vary along the follicle development/growth to exert timely, distinct, required physiological functions. Based on that, we then hypothesized that in rodents, YAP target genes contribute to the initial follicle growth (involving high GC proliferation rates) until the follicles become gonadotropin-dependent, and therefore, require transitory YAP nuclear export for the final maturation/differentiation of the follicle until the return of this Hippo effector to the nucleus, where its transcriptional activity is critical for ovulation. Interestingly, it seems that the same hypothesis proposed for rodents can also be proposed for monovulatory species, particularly for the model employed herein, bovine.

In a recent study by our research group, we demonstrated by in vitro and in vivo approaches that YAP transcriptional activity in preovulatory bovine GC is critical for the LH-induced ovulation in this species [36]. These findings indicate that, in large bovine dominant preovulatory follicles (≥12 mm), YAP must remain unphosphorylated and transcriptionally active in the nucleus to allow LH-induced pre-ovulatory signaling. The present study, nevertheless, shows strong evidence that during the follicle divergence and establishment of dominance, FSH increases YAP phosphorylation to allow the future dominant follicle to increase or accelerate its GC estrogenic capacity. Once the dominance is established, it is most likely that the YAP phosphorylation status returns to basal levels in the periovulatory period. To confirm this latter hypothesis, an ongoing investigation of our group is assessing YAP-TEAD target genes’ expression patterns at later time points along the follicle wave in vivo and, most importantly, we are also evaluating the effects of insulin-like growth factor 1 (IGF1) on YAP activity in bovine GC in vitro. One of the reasons by which the selected dominant follicle continues its growth is directly related to the IGF system. IGF1 increases the sensitivity of small follicles (around 5 mm in cattle) to gonadotropins and simulates their transition from the gonadotropin-responsive to the gonadotropin-dependent stages [37]. This growth factor not only induces E2 secretion in GC, but also synergizes with FSH to promote final differentiation of GC until the luteinizing hormone (LH) surge, which is required for ovulation of the mature dominant follicle [38].

In summary, we provided novel evidence that YAP-TEAD-related transcriptional activity plays an important role in the molecular mechanisms that drive the GC differentiation into a more estrogenic profile during follicular divergence and the establishment of follicular dominance. By regulating YAP activity in bovine granulosa cells, FSH alters the expression of *CTGF* and other classic YAP-TEAD target genes and contributes to the augmented estrogenic capacity of the selected dominant follicle.

## 4. Material and Methods

### 4.1. In Vitro Studies

The reagents used for in vitro cultures were obtained from Thermo Fisher Scientific, except where otherwise stated. The granulosa cell (GC) culture employed herein is a completely serum-free, long-term GC culture system, also described as a GC differentiation culture system [18,31]. In such conditions, GC are responsive to FSH and maintain an estrogenic phenotype with a minimum of luteinization along the culture [39,40]. Briefly, bovine ovaries were collected in local abattoirs from random adult cows and were transported to the laboratory in PBS containing penicillin (100 IU/mL) and streptomycin (100 μg/mL). Follicles between 2 and 5 mm in diameter were dissected from the ovarian stroma and sectioned in Dulbecco’s Modified Eagle Medium Nutrient Mixture F-12 (DMEM/F12). GC were then collected by rinsing the follicle walls with DMEM/F12, washed twice by centrifugation at 980× *g* for 20 min each, and filtered through a Cell Dissociation Sieve—Tissue Grinder Kit/150 Mesh (Sigma-Aldrich, Oakville, ON, Canada). Finally, GC were suspended in basal culture media composed by DMEM/F12 supplemented with sodium bicarbonate (10 mM), sodium selenite (4 ng/mL), BSA (1 mg/mL), penicillin (100 IU/mL), streptomycin (100 μg/mL), human transferrin (5 ng/mL), non-essential amino acid mix (10 mM), androstenedione (A4; 10^−7^ M at start of culture, and 10^−6^ Mat each medium change), and insulin (10 ng/mL). The number of cells was counted with a hemocytometer and the viable cells were assessed by the dye exclusion method using 0.4% Trypan Blue. For cultures, GC were seeded into 24-well tissue culture plates (Sarstedt Inc., St-Leonard, QC, Canada) at a density of 1 × 10^6^ viable cells per well in 1 mL of medium. Cultures were maintained at 37 °C in 5% CO_2_ in air for 6 days with 70% (700 μL) medium being replaced every 2 days and treatments added from day 2 on (on days 2 and 4 of culture). Although insulin (10 ng/mL) was added since day 0 and at each medium change (day 2 and day 4), depending on the experiment, cells were also treated for the last 4 days of culture with human FSH (1 or 10 ng/mL) or with distinct concentrations of the pharmacological inhibitors Verteporfin (VP; Sigma-Aldrich) or Peptide 17 (P17; Selleck Chemicals, Houston, TX, USA). Medium samples were collected on day 6 and stored at −20 °C until the steroid assay, and cells were collected on day 6 in Trizol or M-PER^®^ mammalian protein extraction reagent and stored at −80 °C until RNA or protein extraction, respectively. All series of cultures were performed on at least three different pools of cells collected on different occasions.

#### 4.1.1. Steroid Assay

Estradiol (E2) was measured from culture media samples collected on day 6 of culture. The concentration was determined by a chemiluminescence kit (ADVIA Centaur, Siemens, Munich, Germany) in a specialized clinical analysis laboratory following the manufacturer’s recommendations.

#### 4.1.2. Western Blotting

Total protein from GC was extracted using M-PER^®^ mammalian protein extraction reagent according to the manufacturer’s instructions and protein levels were quantified using the Pierce™ BCA Protein Assay Kit. Halt™ Protease and Phosphatase Inhibitor Cocktails were added to the samples’ final solutions to avoid protein degradation. Samples (20–40 μg) were resolved on 12% sodium dodecyl sulfate-polyacrylamide gels and transferred to Hybond-P PVDF membrane (GE Amersham, Mississauga, ON, Canada). Membranes were then probed at 4 °C overnight in 5% BSA in TTBS with different primary antibodies (details and dilutions for each antibody are indicated in Table 1). After washing three times with TTBS, membranes were incubated for 1 h at room temperature with anti-rabbit HRP-conjugated IgG diluted in 5% non-fat dry milk in TTBS. Protein bands were visualized by chemiluminescence (ECL; Millipore, Billerica, MA, USA) and quantified using a ChemiDoc MP detection system (Bio-Rad, Hercules, CA, USA) and Image Lab™ software.

### 4.2. Ex Vivo Study

The ex vivo study used reagents obtained from Thermo Fisher Scientific (Saint-Laurent, QC, Canada), except where otherwise stated.

#### 4.2.1. Tissue Sampling

Bovine ovaries were collected on different days from random adult cows at a local abattoir and were transported to the laboratory in PBS at 35 °C containing penicillin (100 IU/mL), streptomycin (100 μg/mL), and fungizone (1 μg/mL). At least five ovaries from different animals that each contained small (<5 mm) and medium (5–10 mm) follicles concomitantly (compatible with those found in emergence to follicle dominance) were selected for further analysis.

#### 4.2.2. Immunohistochemistry

For immunohistochemistry (IHC) evaluation, bovine ovaries were selected as described above. Entire ovaries were then fixed in 10% formaldehyde solution for 24 h, rinsed, and dehydrated in alcohol until they were embedded in paraffin. Serial sections were prepared (at a thickness of 3 µm), followed by deparaffinization, rehydration, sodium citrate heat-mediated antigen retrieval, peroxidase block, and protein blocking (10% goat for 30 min), and then slides were probed with primary antibody against total and phosphorylated forms of YAP (Table 1) overnight at 4 °C. Protein detection was then performed with the Vectastain Elite ABC HRP Kit (VECTPK6101, Vector Laboratories, Burlingame, CA, USA) and stained with the DAB substrate kit (VECTSK4100, Vector Laboratories). Slides were then counterstained with hematoxylin and dehydrated with graded alcohols prior to mounting. Negative controls were included in the IHC analysis and consisted of slides for which the primary antibodies (for both total and phosphorylated YAP) were omitted. The results confirmed the specificity of our second antibody (not shown). Photomicrographs were taken using a Carl Zeiss Axio Imager M1 microscope (Carl Zeiss, Toronto, ON, Canada) at ×1000 magnification and using the Zen 2012 blue edition software (Carl Zeiss).

### 4.3. In Vivo Study

The reagents used for the in vivo experiment were obtained from Sigma-Aldrich Co., except where otherwise stated. All experimental procedures using cattle were reviewed and approved by the Federal University of Santa Maria Animal Care and Use Committee (ACUC No. 23081.009594/2007-41). To obtain GC from the largest (F1) and the second largest (F2) follicles (also referred to herein as dominant and subordinate follicles, respectively), ovaries were collected from the first follicular growth wave of the estrous cycle. For this, thirty-six weaned beef cows (predominantly Hereford and Aberdeen Angus) were injected with two doses of PGF2α analogue (Cloprostenol, 125 µg; Schering-Plough Animal Health, Kenilworth, NJ, USA) intramuscularly (i.m.), 12 h apart. They were then observed in estrus within 3–5 days after PGF2α. Ovaries were then examined once a day by transrectal ultrasonography, using an 8 MHz linear-array transducer (Aquila Vet scanner, Pie Medicals, Maastricht, The Netherlands), and all follicles larger than 5 mm were drafted using three to five virtual slices of the ovary, allowing a three-dimensional localization of follicles and monitoring individual ovarian follicles’ location during the follicular wave [3]. The day of the follicular emergence was designated as day 0 (D0) of the wave and it was retrospectively identified as the last day on which the dominant follicle was 4 or 5 mm in diameter [8]. The cows were then randomly assigned to be ovariectomized by colpotomy at days 2 (D2), 3 (D3), or 4 (D4) of the follicular wave (four cows per group for each day) to recover the largest (F1: herein also referred to as dominant follicle) and the second largest (F2: herein also referred to as subordinate follicle) follicles from each cow. After ovariectomy, GC were recovered from F1 and F2 follicles and stored at –80 °C until RNA extraction for RT-qPCR analysis.

### 4.4. RNA Extraction, Reverse Transcription, and Quantitative PCR (qPCR) for In Vitro and In Vivo Studies

Total RNA from in vitro culture samples was extracted using the PureLink™ RNA Mini Kit according to the manufacturer’s instructions. Total RNA from the in vivo samples was extracted using the silica column-based protocol (Qiagen, Mississauga, ON, Canada) according to the manufacturer’s instructions. For reverse transcription reaction (RT), total RNA (0.2 μg from both in vitro and in vivo samples) was first treated with 1U DNase (Promega, Madison, WI, USA) at 37 °C for 5 min to digest any contaminating DNA. The RNA was then reverse-transcribed in the presence of 1 mM of oligo (dT) primer and 4U Omniscript Rtase (Qiagen), 0.25 mM of dideoxy-nucleotide triphosphate (dNTP) mix, and 19.33U RNase Inhibitor (GE Healthcare, Chicago, IL, USA) in a volume of 20 μL at 37 °C for 1 h. The reaction was terminated by incubation at 93 °C for 3 min. Real-time PCR was conducted in an ABI Prism 7300 instrument in a 25 μL reaction volume containing 12.5 μL of 2 × Power SYBR Green PCR Master Mix (Applied Biosystems, Waltham, MA, USA), 9.5 μL of water, and 1 μL of each sample cDNA and bovine-specific primers (Table 2). Cycling conditions were 3 min at 95 °C, followed by 40 cycles of 15 s at 95 °C, 30 s at 60 °C, and 30 s at 72 °C. In each run, melting curve analysis was used to verify that a single product was amplified. Each reaction was performed in duplicate, and the average threshold cycle (Ct) value was used to calculate relative mRNA abundance of target genes relative to the housekeeping genes *H2AFZ* (for in vitro samples) and *GAPDH* (for in vivo samples) and with the 2−∆∆Ct method and correction for amplification efficiency [41]. Primers not published previously were designed based on sequences from GenBank, using the Primer-BLAST platform, and their respective amplicons were sequenced to confirm their specificity.

### 4.5. Statistical Analysis

The statistical analyses for all experiments were performed using JMP Software (SAS Institute Inc., Cary, NC, USA). Data that were not normally distributed (Shapiro–Wilk test) were transformed to natural logarithms. For mRNA abundance or target protein levels, ANOVA was used to test for the main effect (treatment) and culture replicate was included as a random effect. Multiple comparisons were tested using the Tukey–Kramer honestly significant difference (HSD) test to compare all treatment groups within the same experiment. All data were presented as means ± SEM and variables were considered statistically significant at *p* < 0.05, represented with different letters. For the in vivo experiment, the day-match differences in continuous data between the dominant (F1) and the subordinate (F2) were assessed by a paired Student’s t test using the cow as the subject. The in vivo data were presented as means ± SEM and variables were considered statistically significant at *p* < 0.05, represented with an asterisk symbol (*).

## Figures and Tables

**Table 1:** List of antibodies used for IHC and WB.

Name of Antibody	Manufacturer (Cat. No.)	Type	Dilution WB	Dilution IHC
ß-actin (C4)	Santa Cruz (sc-47778 HRP)	CkM	1:10,000	
YAP (D8H1X)	Cell signaling (14074)	RbM	1:1000	1:250
Phospho-YAP (Ser127) (D9W2I)	Cell signaling (13008)	RbM	1:1000	1:250
Anti-Rabbit IgG–HRP Conjugate	Promega (W401B)	Rb	1:1000	

CkM: Chicken monoclonal; RbM: rabbit monoclonal; Rb: rabbit.

**Table 2:** Sequences of primers used in the expression analysis of target genes.

Gene	Sequence 5′→3′	Accession Number
*ANKRD1*	F: ATCAGTGCGCGGGATAAGTT	NM_001034378.2
	R: GGGAGTATCTCCTTCCCGGT	
*CTGF*	F: AGCTGAGCGAGTTGTGTACC	[42]
	R: TCCGAAAATGTAGGGGGCAC	
*CYP19A1*	F: CTGAAGCAACAGGAGTCCTAAATGTACA	[43]
	R: AATGAGGGGCCCAATTCCCAGA	
*CYR61*	F: GGCTCCCCGTTTTGGAATG	NM_001034340.2
	R: TCATTGGTAACGCGTGTGGA	
*GAPDH*	F: GATTGTCAGCAATGCCTCCT	[36]
	R: CGTTCTCTGCCTTGACTGTG	
*H2AFZ*	F: GAGGAGCTGAACAAGCTGTTG	[43]
	R: TTGTGGTGGCTCTCAGTCTTC	

Forward (F) and reverse (R) primers used in RT-qPCR.

## Data Availability

Not applicable.
